# Pregnant Women With Ulcerative Colitis Have a Higher Risk of Delivering Small‐For‐Gestational‐Age Infants: The Japan Environment and Children's Study (JECS)

**DOI:** 10.1111/jog.70109

**Published:** 2025-10-11

**Authors:** Tamami Tsuzuki, Marina Minami, Ryuhei Nagai, Yusuke Oki, Hedeyuki Miyachi, Masamitsu Eitoku, Narufumi Suganuma, Nagamasa Maeda, The Japan Environment and Children's Study Group, Michihiro Kamijima, Shin Yamazaki, Maki Fukami, Reiko Kishi, Chiharu Ota, Koichi Hashimoto, Chisato Mori, Shuichi Ito, Ryoji Shinohara, Hidekuni Inadera, Takeo Nakayama, Ryo Kawasaki, Yasuhiro Takeshima, Seiji Kageyama, Narufumi Suganuma, Shoichi Ohga, Takahiko Katoh

**Affiliations:** ^1^ Department of Obstetrics and Gynecology, Kochi Medical School Kochi University Nankoku Kochi Japan; ^2^ Integrated Center for Advanced Medical Technologies (ICAM‐Tech) Kochi Medical School Hospital Nankoku Kochi Japan; ^3^ Department of Gastroenterology and Hepatology, Kochi Medical School Kochi University Nankoku Kochi Japan; ^4^ Department of Environmental Medicine, Kochi Medical School Kochi University Nankoku Kochi Japan

**Keywords:** inflammatory bowel disease, JECS study, pregnancy, small‐for‐gestational‐age, ulcerative colitis

## Abstract

**Aim:**

Ulcerative colitis primarily affects individuals of reproductive age, raising concerns regarding its impact on pregnancy, lactation, and postpartum outcomes. Although numerous international studies exist, research on the Japanese population remains limited. This study aimed to analyze data from a large‐scale nationwide cohort in Japan.

**Methods:**

Data were derived from a prospective birth cohort study conducted between 2011 and 2014 that recruited pregnant women in early pregnancy across Japan. Of the 97 075 pregnancies, 214 were complicated by ulcerative colitis. These were compared with 96 861 pregnancies without a history of ulcerative colitis in terms of background characteristics, perinatal outcomes, and neonatal findings.

**Results:**

Pregnant women with ulcerative colitis were older and had a lower smoking rate than those without the condition. Gestational weight gain was lower in the ulcerative colitis group than in the control group. The proportion of small‐for‐gestational‐age infants was higher in pregnancies complicated by ulcerative colitis. This risk is further elevated in patients with anemia or inflammation during early pregnancy.

**Conclusions:**

There is limited research on the impact of ulcerative colitis on pregnancy in Japan. This study found that pregnancies complicated by ulcerative colitis were associated with a higher risk of delivering small‐for‐gestational‐age infants. Further studies with more detailed data on disease status are needed to assess disease activity and perinatal risk better.

## 1 Introduction

Ulcerative colitis (UC) is an inflammatory bowel disease (IBD) with a peak onset age of 15–30 years, followed by another peak at 50–70 years [1]. The incidence of UC varies by region, being higher in Northern Europe and North America and relatively lower in Asia, including Japan [2].

Since the onset of UC often coincides with reproductive age, concerns regarding its impact on pregnancy have emerged. Although several studies have addressed this issue, most have been conducted outside of Japan. These studies reported varying effects of UC on maternal and neonatal outcomes during pregnancy and postpartum. Some studies suggest that UC does not increase perinatal risk [3], whereas others indicate an elevated risk of preterm birth and low birth weight [4, 5]. Active disease during pregnancy is associated with an increased risk of preterm birth, low birth weight, and cesarean delivery [4, 6, 7].

Although the incidence of UC was once considered low, it has recently increased in Asia, including Japan. The symptoms of UC vary by ethnicity, and their impact on pregnancy may differ according to racial background. Therefore, accumulating data on UC in the Japanese and other Asian populations is crucial.

Crohn's disease (CD), another major type of IBD, accounts for only 35 cases (0.04%) in the Japan Environment and Children's Study (JECS), a large‐scale cohort study conducted nationwide in Japan between 2011 and 2014. Although UC and CD share overlapping symptoms and are both classified as IBD, they differ in terms of the affected sites, disease pathology, and reported effects on perinatal outcomes [3].

Given these differences and the limited number of CD cases in the dataset, CD was considered a distinct condition and was excluded from this study. We aimed to analyze data from the JECS, focusing on the impact of UC on perinatal complications and neonatal outcomes. Our analysis was limited to patients with UC.

## 2 Methods

### 2.1 Study Design and Settings

The JECS is a prospective cohort study funded by the Japanese Ministry of the Environment that primarily investigates environmental factors affecting children's health and development. Between January 2011 and March 2014, approximately 100 000 pregnant women from 15 Regional Centers in Japan were recruited. The eligibility criteria included plans to remain in Japan and sufficient proficiency in Japanese. Participants were followed up from around the 10th week of pregnancy until their children reached 13 years of age. Detailed reports on JECS have been published [8, 9].

For this analysis, we used the jecs‐ta‐20 190 930 dataset released in October 2019, which included data until the children reached 3 years of age. Information was obtained from the self‐administered questionnaires and medical record transcripts.

### 2.2 Participants

Among the pregnant women in JECS, those with multiple pregnancies were excluded. Women with unknown birth weights or unclear information on live births, stillbirths, or miscarriages were excluded. To identify the risk factors for small‐for‐gestational‐age (SGA) infants, the UC group was further divided into SGA and non‐SGA.

The study was conducted in accordance with the principles of the Declaration of Helsinki. The JECS protocol was reviewed and approved by the Ministry of the Environment's Institutional Review Board for Epidemiological Studies (no. 100910001) and the Ethics Committees of all participating institutions. Written informed consent was obtained from all participants.

### 2.3 Variables

The primary outcome was whether the newborn was SGA, defined as a birth weight below the 10th percentile for the gestational age [10]. UC was the exposure variable, identified through a questionnaire listing gastrointestinal conditions, including UC. Women who added a check for UC in their questionnaires were classified into the UC group, and those who did not have a check for UC in their questionnaires were assigned to the control group.

Maternal age at delivery was categorized into six groups (≤ 19, 20–24, 25–29, 30–34, 35–39, and ≥ 40 years). Pre‐pregnancy body mass index (BMI) was categorized as underweight (BMI < 18.5), normal‐weight (18.5 ≤ BMI < 25), or obese (BMI ≥ 25). The conception methods included natural conception, ovulation induction, artificial insemination, and assisted reproductive technology (ART). Smoking status was classified as “Never,” “Previously did, but quit before realizing current pregnancy,” “Previously did, but quit after realizing current pregnancy,” or “Currently smoking.” Alcohol consumption during pregnancy was categorized as present or absent.

Educational attainment was classified as compulsory, high school graduate, or higher. Household income was categorized as < 2 million, 2–6 million, 6–10 million, or > 10 million yen. Gestational age at delivery was calculated based on the last menstrual period or crown‐rump length at 8–10 weeks for natural conceptions and treatment dates for infertility treatments. The delivery mode was categorized as vaginal or cesarean section.

Gestational weight gain was calculated as the difference between pre‐pregnancy weight and weight at delivery, classified as insufficient, appropriate, or excessive, based on the Japan Society of Obstetrics and Gynecology (JSOG) 2021 guidelines [11]. The guidelines define appropriate weight gain as 12–15 kg for underweight women (BMI < 18.5), 10–13 kg for normal‐weight women (BMI 18.5–25.0), and 7–10 kg for obese women (BMI ≥ 25). In this study, obese women (BMI ≥ 25) were not further subdivided. Energy intake was divided into tertiles (low, normal, and high) [12]. Maternal comorbidities included pre‐pregnancy heart disease, kidney disease, diabetes, or hypertension. Neonatal abnormalities include chromosomal and congenital malformation.

Preterm birth was defined as delivery at 22–36 weeks of gestation. Perinatal complications included preterm birth, stillbirth, premature rupture of membranes, gestational diabetes, hypertensive pregnancy, placental abnormalities, and placental abruption.

Apgar scores at 1 and 5 min were categorized as severe asphyxia (0–3 points) or mild asphyxia (4–7 points). Umbilical artery blood gas pH and placental weight were analyzed as continuous variables.

Early pregnancy blood test results were used to explore the relationship between UC severity and SGA status. Hemoglobin level (cut‐off: 10 g/dL) indicated anemia, and platelet count (cut‐off: 260000/μL) indicated inflammation [13]. These data were only available in early pregnancy.

Data on UC and smoking history were obtained from the questionnaires administered during early pregnancy. Data on alcohol consumption, household income, education level, and energy intake were obtained from questionnaires administered during the second/third trimester. Data on gestational age, maternal weight, conception method, delivery method, perinatal complications, maternal complications, and neonatal findings were obtained from medical record transcripts. Non‐fasting maternal blood samples were collected by medical staff when the pregnant women visited co‐operating health care providers during periods named in JECS as MT1 (gestational age 12–16 weeks). Blood samples were sent to a central laboratory within 48 h and were assayed by a commercial laboratory (SRL Inc., Tokyo). Hemoglobin (Hb) levels were measured using the SLS‐Hemoglobin method with the SYSMEX XE‐2100, and platelet (Plt) counts were determined using hydrodynamic focusing DC detection with the SYSMEX XE‐2100 [14].

### 2.4 Data Analysis

Data were analyzed using the Stata software (version SE 17; StataCorp LLC, College Station, Texas, United States). Descriptive statistics were used to compare maternal characteristics. Continuous variables were analyzed using *t*‐tests, whereas categorical variables were compared using the chi‐square (*χ* ^2^) test. Statistical significance was set at *p* < 0.05.

Logistic regression analysis was performed to evaluate the association between UC and SGA birth. Adjusted odds ratios (ORs) with 95% confidence intervals (CIs) were calculated, adjusting for maternal age, smoking, alcohol consumption, gestational weight gain, energy intake, hypertensive disorders of pregnancy, preterm birth, maternal complications, and fetal congenital anomalies. Subgroup analyses were conducted using early pregnancy hemoglobin levels and platelet counts as indicators of UC severity.

## 3 Results

**FIGURE 1:**
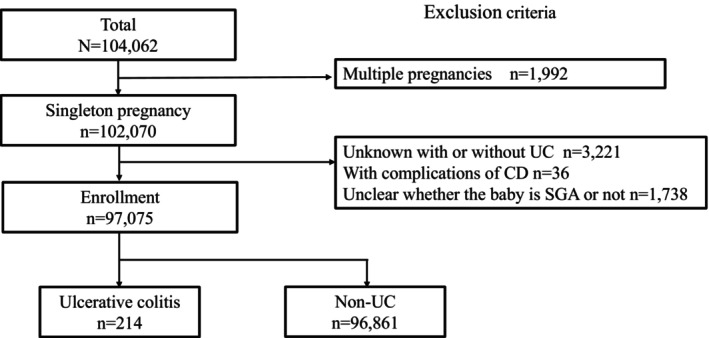
Flowchart of case selection. Of a total of 104 062 cases, exclusions were made based on criteria including multiple pregnancies, unknown ulcerative colitis (UC) status, complications of Crohn's disease (CD), and uncertainty regarding small‐for‐gestational‐age (SGA) status. In total, 97 075 cases were enrolled, comprising 214 cases with ulcerative colitis (UC) and 96 861 cases without UC. CD, Crohn's disease; SGA, small‐for‐gestational‐age; UC, ulcerative colitis.

In total, 104 062 records were included in the JECS, of whom 102 070 were records of singleton pregnancies after excluding those of multiples. Participants with unclear UC status and missing data on fetal development (cases with unknown information on the infant's sex, birth weight, gestational age at delivery, and maternal parity) were excluded. The final analysis included 97 075 pregnant women, of whom 214 had a history of UC, resulting in a prevalence of 0.2% (Figure 1).

The participants' background data are shown in Table 1. UC was significantly less common in the 20–24 year age group. Maternal BMI before pregnancy, categorized as underweight, normal‐weight, or overweight, showed no significant differences between the two groups. Methods of conception, including natural conception, ovulation induction, artificial insemination, and ART, did not differ significantly. The UC group had a higher proportion of non‐smokers than the control group. There were no significant differences in alcohol consumption or household income; however, fewer individuals with lower educational levels were found in the UC group.

**TABLE 1:** Patient backgrounds.

	All	UC	Control	*p*
	*n* = 97 075	*n* = 214	*n* = 96 861	
Age category, *n* (%), years				
≤ 19	836 (0.9)	0 (0)	836 (0.9)	
20–24	8769 (9.0)	8 (3.7)	8761 (9.1)	
25–29	26 783 (27.6)	56 (26.2)	26 727 (27.6)	
30–34	34 407 (35.5)	79 (36.9)	34 328 (35.4)	
35–39	21 831 (22.5)	60 (28.0)	21 771 (22.5)	
≥ 40	4443 (4.6)	11 (5.1)	4432 (4.6)	0.04
BMI category, *n* (%)				
< 18.5	15 653 (16.1)	42 (19.7)	15 611 (16.1)	
18.5–24.9	70 961 (73.1)	156 (73.2)	70 805 (73.1)	
≥ 25.0	10 417 (10.7)	15 (7.0)	10 402 (10.7)	0.11
Method of conception, *n* (%)				
Natural	90 449 (93.6)	201 (94.4)	90 248 (93.6)	
Ovulation induction	2470 (2.6)	7 (3.3)	2463 (2.6)	
AIH	902 (0.9)	1 (0.5)	901 (0.9)	
ART	2842 (2.9)	4 (1.9)	2838 (2.9)	0.62
Smoking, *n* (%)				
Never	56 801 (58.2)	140 (66.7)	55 941 (58.2)	
Previously did, but quit before realizing current pregnancy	22 832 (23.7)	47 (22.4)	22 785 (23.7)	
Previously did, but quit after realizing current pregnancy	12 772 (13.3)	18 (8.6)	12 754 (13.3)	
Current smoker	4666 (4.9)	5 (2.4)	4661 (4.9)	0.03
Alcohol, *n* (%)				
No drinking during pregnancy	48 347 (50.7)	112 (52.8)	48 235 (50.7)	
Drinking alcohol during pregnancy	46 956 (49.3)	100 (47.2)	46 856 (49.3)	0.54
Household Income, *n* (%)				
< 2 000 000 JPY	5063 (5.7)	8 (4.0)	5055 (5.7)	
2 000 000–5 999 999 JPY	60 394 (67.7)	131 (66.2)	60 263 (67.7)	
6 000 000–9 999 999 JPY	19 996 (22.4)	54 (27.3)	19 942 (22.4)	
≥ 10 000 000 JPY	3812 (4.3)	5 (2.5)	3807 (4.3)	0.21
Maternal education, *n* (%)				
Less than high school	4619 (4.8)	3 (1.4)	4616 (4.8)	
High school or equivalent	30 102 (31.5)	58 (27.1)	30.044 (31.5)	
Vocational/associate degree	40 172 (42.0)	97 (45.3)	40 075 (42.0)	
Bachelor's degree or higher	20 656 (21.6)	56 (26.2)	20 600 (21.6)	0.03

*Note:* This table shows background data of the patients, including age, BMI, method of conception, smoking status, drinking status, household income, and educational level. We found predominant differences in age, smoking status, and educational level. Several values were missing for each variable.

Abbreviations: AIH, artificial insemination; ART, assisted reproductive technology; BMI, body mass index; UC, ulcerative colitis.

The pregnancy course and perinatal complications were examined (Table 2). No significant differences were observed between groups in terms of birth weeks, energy intake during pregnancy, or perinatal complications. The cesarean section rate was similar in both groups: 19.7% and 18.8% in the UC and control groups, respectively. There were 13 categories of indications for cesarean section: previous cesarean section, previous uterine surgery, hypertensive disorder of pregnancy, placenta previa, non‐reassuring fetal status, abnormal fetal position, arrest of labor, multiple pregnancy (already excluded), premature rupture of membranes, intrauterine infection, cephalopelvic disproportion, pregnancy beyond the estimated due date (≥ 40 weeks), and complications and others. If UC is an indication for cesarean section, complications and others may be considered relevant. However, the frequency was similar in the UC (3.2%) and control (3.3%) groups (data not shown). Gestational weight gain tended to be lower in pregnant women with UC. In blood tests conducted during early pregnancy, pregnant women with UC had lower hemoglobin levels and higher platelet counts than pregnant women in the control group.

**TABLE 2:** Delivery outcomes and perinatal complications.

	All	UC	Control	*p*
	*n* = 97 075	*n* = 214	*n* = 96 861	
Birth weeks, weeks, average ± SD	39.2 ± 1.6	39.3 ± 1.4	39.22 ± 1.6	0.49
Delivery method, *n* (%)				
Vaginal	78 581 (81.2)	171 (80.3)	78 410 (81.2)	
Cesarian section	18 223 (18.8)	42 (19.7)	18 181 (18.8)	0.74
Energy intake (mid‐pregnancy), *n* (%)				
Low	32 139 (33.4)	62 (29.0)	32 077 (33.5)	
Adequate	32 011 (33.3)	78 (36.5)	31 933 (33.3)	
High	31 945 (33.2)	74 (34.6)	31 871 (33.2)	0.36
Degree of gestational weight gain, *n* (%)				
Insufficient	44 414 (47.2)	111 (53.9)	44.303 (47.2)	
Appropriate	29 487 (31.4)	69 (33.5)	29 418 (31.3)	
Excessive	20 166 (21.4)	26 (12.6)	20 140 (21.5)	< 0.01
Blood test results (early pregnancy), average ± SD				
Hemoglobin, g/dL	12.0 ± 1.0	11.8 ± 1.0	12.0 ± 1.0	< 0.01
Platelet, 10^6^ /μL	24.9 ± 5.2	26.9 ± 6.4	24.9 ± 5.2	< 0.01
Total cholesterol, mg/dL	199.7 ± 34.8	203.9 ± 35.0	199.7 ± 34.8	0.10
Perinatal complications, *n* (%)				
Preterm delivery	4703 (4.8)	7 (3.3)	4696 (4.9)	0.28
Stillbirth	226 (0.2)	0 (0)	226 (0.2)	0.78
Premature rupture of membrane	8038 (8.3)	22 (10.3)	8016 (8.3)	0.29
Gestational diabetes mellitus	2616 (2.7)	4 (1.9)	2612 (2.7)	0.46
Hypertensive disorders of pregnancy	3003 (3.1)	3 (1.4)	3000 (3.1)	0.15
Placenta abnormal	24 (0.02)	0 (0)	24 (0.02)	0.82
Abruption	425 (0.4)	1 (0.5)	424 (0.4)	0.95

*Note:* This table presents data on birth outcomes, including the number of weeks of delivery, method of delivery, energy intake (mid‐pregnancy), weight gain during pregnancy, blood test results during early pregnancy, and perinatal complications. Significant differences were found in the degree of weight gain during pregnancy, hemoglobin levels, and platelet counts. Several values were missing for each variable.

Abbreviations: SD, standard deviation; UC, ulcerative colitis.

Delivery and neonatal outcomes are shown in Table 3. There were no significant differences in birth weight, Apgar scores at 1 and 5 min, umbilical‐cord blood pH, or placental weight, except for a higher incidence of SGA in the UC group.

**TABLE 3:** Neonatal outcomes and birth characteristics.

	All	UC	Control	*p*
	*n* = 97 075	*n* = 214	*n* = 96 861	
Birth weight, *g*, average ± SD	3020.3 ± 427.0	3030.4 ± 412.6	3020.2 ± 427.0	0.73
Birth height, cm, average ± SD	48.9 ± 2.4	49.0 ± 2.2	48.9 ± 2.4	0.37
SGA, *n* (%)	7653 (7.9)	26 (12.2)	7627 (7.9)	0.02
Apgar score at 1 min, *n*(%)				
≤ 3	687 (0.7)	1 (0.5)	686 (0.7)	
4–6	3538 (3.7)	14 (6.8)	3524 (3.7)	
≥ 7	90 496 (95.5)	191 (92.7)	90 305 (95.6)	0.06
Apgar score at 5 min, *n*(%)				
≤ 3	262 (0.3)	1 (0.5)	261 (0.3)	
4–6	907 (1.0)	1 (0.5)	906 (1.0)	
≥ 7	91 022 (98.7)	203 (99.0)	90 819 (98.7)	0.67
Umbilical artery pH, average ± SD	7.312 ± 0.121	7.304 ± 0.065	7.312 ± 0.121	0.40
Placental weight, *g*, average ± SD	559.5 ± 116.6	556.2 ± 98.3	559.5 ± 116.6	0.69

*Note:* This table shows the birth‐related characteristics of newborns, including birth weight, height, Apgar score, umbilical artery pH, and placental weight. Several values were missing for each variable. SGA infants are significantly more common in patients with UC.

Abbreviations: LGA, large for gestational age; SD, standard deviation; SGA, small‐for‐gestational‐age; UC, ulcerative colitis.

Logistic regression analysis was used to assess the association between UC and SGA (Table 4). Confounding factors, including maternal age, smoking, alcohol consumption, gestational weight gain, energy intake, hypertensive disorders of pregnancy, preterm birth, maternal complications, and fetal congenital anomalies, were adjusted in the risk ratio calculation. The adjusted OR was 1.65 (95% CI: 1.07–2.54), indicating that UC significantly increased the risk of SGA.

**TABLE 4:** Odds ratio for UC and SGA.

Variables	COR	95% CI	AOR	95% CI
UC	**1.62**	**1.07–2.44**	**1.65**	**1.07–2.54**

*Note:* This table presents ORs and 95% confidence intervals showing the association between UC and SGA. To examine the effect of UC, we performed a multivariate analysis, adjusting for confounding factors such as maternal age, smoking, alcohol consumption, weight gain during pregnancy, energy intake, hypertensive disorders of pregnancy, preterm birth, maternal complications, and fetal congenital anomalies. The AOR was calculated. Variables with significant differences are indicated in bold.

Abbreviations: AOR, adjusted odds ratio; CI, confidence interval; COR, crude odds ratio; ORs, odds ratios; UC, ulcerative colitis.

Next, we examined whether symptoms during pregnancy affect the risk of SGA in pregnant women with UC. In the JECS, data on subjective symptoms such as bloody stool or increased bowel movements and objective findings such as rectal mucosal inflammation or ulcerative lesions were not collected. Although previous reports were not based on pregnant women, we tentatively estimated the presence or absence of UC symptoms using hemoglobin levels and platelet counts obtained in early pregnancy. A hemoglobin level below 10 g/dL was considered indicative of symptoms, as anemia may progress with signs such as bloody stools. A platelet count above 260 000/μL was considered indicative of inflammation based on reports showing that platelet levels increase with intestinal mucosal inflammation [13]. Pregnant women with UC were divided into two groups: those with hemoglobin levels above 10 g/dL and platelet counts below 260 000/μL (no‐symptoms group) and those with other values (symptoms‐present group). The non‐UC group was used as a reference for comparison. Confounding factors, including maternal age, BMI, smoking, drinking, gestational weight gain, energy intake, gestational hypertension, preterm birth, maternal comorbidities, and fetal congenital anomalies, were adjusted.

The results showed no significant risk of SGA in the no‐symptoms group; however, in the symptoms‐present group, the adjusted OR was 1.94 (95% CI: 1.05–3.59), indicating a significant increase in the risk of SGA compared to the non‐UC group (Table 5).

**TABLE 5:** Risk of SGA by blood test.

	COR	95% CI	AOR	95% CI
non‐UC	Reference		Reference	
UC without symptoms	1.09	0.53–2.25	1.2	0.58–2.48
UC with symptoms	**2.05**	**1.16–3.61**	**1.94**	**1.05–3.59**

*Note:* Hemoglobin levels and platelet counts were used to separate UC into symptomatic (*n* = 94) and non‐symptomatic (*n* = 94) groups and to compare the risk of SGA with that of pregnant women with non‐UC (26 cases of UC with missing blood collection data). The risk of SGA did not increase in the non‐symptomatic group, but the risk of SGA increased in the symptomatic group. As in Table 4, the AOR was calculated after adjusting for confounding factors, including maternal age, smoking, alcohol consumption, weight gain during pregnancy, energy intake, hypertensive disorders of pregnancy, preterm birth, maternal complications, and fetal congenital anomalies. Variables with significant differences are indicated in bold.

Abbreviations: AOR, adjusted odds ratios; CI, confidence interval; COR, crude odds ratio; UC, ulcerative colitis.

## 4 Discussion

Pregnant women with UC were older and had a lower smoking rate than those without the condition. Gestational weight gain was also lower, and the proportion of SGA infants was higher in pregnancies that were complicated by UC. The risk of SGA may further increase in patients with UC with anemia or inflammation during early pregnancy.

UC and CD are representative forms of IBD. Although immunological abnormalities are suspected to contribute to IBD, the exact cause remains unclear. These diseases are characterized by flare‐ups and remission, with no complete cure. UC primarily affects the mucosal layer of the colon, causing nonspecific inflammation that leads to erosion and ulcers.

UC is diagnosed based on symptoms such as recurrent bloody diarrhea, mucus‐streaked stools, and characteristic findings from endoscopy, barium enema radiography, and histopathological analyses. The disease has an active phase, with symptoms and endoscopic findings, and a remission phase, with no symptoms or findings. Achieving and maintaining remission through drug therapy is essential to improve patients' quality of life. This highlights the ongoing challenges in managing UC, and the importance of monitoring and controlling the disease to prevent flare‐ups and to maintain long‐term remission.

The global prevalence of IBD is 0.3% [2]; however, regional differences exist, with higher rates in Western countries [15]. Systematic reviews report an annual incidence of UC in Europe of approximately 24.3 per 100 000 people [16]. Although IBD was traditionally rare in Asia, cases have increased, particularly in Japan, South Korea, China, and India [17, 18, 19]. Environmental factors, such as lifestyle and diet, are believed to have contributed to this rise [17]. In multiethnic countries such as Singapore and Malaysia, the incidence varies by ethnicity, with Indians having higher rates than Chinese and Malays, suggesting racial differences [20, 21]. Although genetic loci related to IBD have been identified, genetic factors alone do not account for its onset [22]. IBD results from the interactions between genetic and environmental factors, leading to inappropriate immune responses in the colonic mucosa [23].

IBD typically affects individuals aged 10–30 years old. The relationship between pregnancy and IBD has been extensively studied. IBD is more common in UC than in CD [24]. During pregnancy, flare‐ups may not occur if the disease is in remission. However, if active, two‐thirds of women experience persistent activity, and another two‐thirds experience exacerbation [24, 25]. In UC, up to 45% of pregnancies during the active phase result in symptom worsening [26]. Treatments for IBD include 5‐aminosalicylic acid (5ASA), thiopurines, steroids, immunosuppressive agents, and biologics. Some medications are contraindicated during pregnancy, requiring women with IBD who wish to conceive to consider drug use carefully. While dietary therapy for IBD lacks clear necessity [27], evidence suggests the importance of adequate energy intake, vitamin supplementation, smoking cessation, and alcohol avoidance [28, 29]. Additionally, iron supplementation is recommended for IBD patients with anemia [30]. Fertility rates during remission are similar to those of healthy individuals [29]; however, infertility is more common in active disease [31]. Pelvic surgery for UC may cause infertility due to adhesions affecting tubal passage [32]. Sulfasalazine is associated with a reversible decrease in sperm motility in men.

The impact of IBD on pregnancy has been studied in three areas: the effect of the disease on pregnancy, the influence of disease severity, and the impact of pharmacological treatments. Studies have reported an increased risk of miscarriage, preterm birth, and low birth weight in women with IBD [4]. Stephansson et al. found that pregnant women with UC had a higher risk of preterm birth, SGA infants, neonatal death, and cesarean sections [5]. A study of placental pathology showed that IBD is associated with maternal vascular malformations and inflammatory changes in the placenta, correlating with a higher incidence of SGA [33]. However, some studies have found no significant effect of IBD on perinatal outcomes, particularly in women with IBD in remission [3].

The relationship between disease activity and pregnancy outcomes was explored. As previously mentioned, studies have shown that when IBD activity is high, the risk of preterm birth, SGA, neonatal death, and cesarean section increase [5, 6, 7]. However, disease activity in IBD does not necessarily influence perinatal complications [34], suggesting that disease control through remission is crucial for pregnancy outcomes.

Patients with IBD should achieve and maintain remission before and throughout pregnancy and breastfeeding, and pharmacological treatment is crucial. Medications such as methotrexate and thalidomide should be avoided because of the risk of fetal harm. Generally, continuing with other treatments during pregnancy does not negatively affect perinatal outcomes.

Most studies on IBD and pregnancy have been conducted in Europe and the United States, with few reports from Japan [35, 36]. Racial and genetic differences are crucial for understanding IBD pathophysiology. Examining the impact of IBD on pregnancy in Asian populations is vital because of the increasing number of IBD cases in this region.

Women with UC in the predominantly Japanese JECS cohort exhibited an increased risk of delivering SGA infants compared with controls, but no significant differences in preterm birth or cesarean section rates were observed. Regarding methods of delivery, cesarean section may be chosen if there are anal lesions. However, it was not clear from the data whether UC was an indication for cesarean section. These findings may be influenced by racial factors, although Japan's exceptional maternal and child health services could potentially alleviate IBD‐related pregnancy risks.

We aimed to identify the group with potentially severe UC symptoms using laboratory data due to the absence of subjective symptoms data and objective findings related to UC in this cohort. The Japanese guidelines for UC indicate a hemoglobin level below 10 g/dL as a marker of severity. Additionally, a platelet count of 260 000/μL or higher was used to assess endoscopic activity in UC patients [13]. Some patients exhibit endoscopic signs, such as ulcers or mucosal inflammation, even without symptoms. Pregnant women in endoscopic remission face a low perinatal risk [37].

Anemia was defined as a hemoglobin level below 10 g/dL, and inflammation was indicated by a platelet count above 260 000/μL as a marker for UC. Patients with no or mild anemia and normal platelet counts were deemed to have stable UC, whereas those with severe anemia or high platelet counts, indicating mucosal inflammation, suggested active symptoms. The study found no significant difference in the incidence of SGA infants in the stable group; however, the active group showed a higher risk of delivering SGA infants than pregnancies without UC. These findings imply that active disease symptoms or endoscopic markers in UC may increase the risk of SGA infants, underscoring the need for symptom control before pregnancy to mitigate this risk in women with UC.

Although colonoscopy is typically avoided during pregnancy, blood tests can offer a noninvasive method to monitor UC in pregnant women. While hemoglobin levels and platelet counts from general UC populations might not be directly applicable to pregnant women, they can still be useful starting points for creating noninvasive biomarkers to track disease activity during pregnancy.

This speculative study calls for more cases and detailed analysis to understand the impact of disease severity on SGA in Japanese women with UC. Developing a reliable and minimally invasive method to assess disease activity in pregnant patients with UC is essential for better management and outcomes.

This study analyzed data from approximately 100 000 pregnant women in Japan, representing the first large‐scale investigation of UC in this demographic. With the anticipated increase in IBD cases, this study offers crucial data on the prevalence of UC among pregnant Japanese women. This information will become increasingly significant as the number of pregnancies complicated by UC is expected to increase.

Analyzing UC using JECS data has several limitations: the basis for UC diagnosis is unclear, detailed medication and treatment history are lacking, UC severity cannot be assessed, and blood test data (hemoglobin level and platelet count) are only available in early pregnancy. The presence of UC was based on self‐reported questionnaires from pregnant women, leaving uncertainty regarding diagnosis and management. There were no questions about subjective symptoms, such as stool frequency or characteristics, or detailed medication information, and objective findings, such as ulcer and inflammation of rectal mucosa and anal lesions. Ideally, the disease status of UC during pregnancy (whether remission was maintained or disease severity changed) should be determined based on these symptoms and findings. The symptoms of UC were assessed using surrogate markers derived from blood test results, such as hemoglobin levels and platelet counts; however, these data were available only in early pregnancy and thus did not permit the evaluation of changes throughout the course of pregnancy.

Birth size was defined using the Japanese neonatal birth weight standards, which may vary with different population standards. Additionally, women with less favorable pregnancy outcomes may have been excluded, potentially underestimating the associated risks.

To understand the effect of UC on pregnancy outcomes and neonatal health, its pathophysiology must be further elucidated. A nationwide cohort study collecting data on disease activity and treatment regimens in pregnant Japanese women is also necessary to comprehensively investigate perinatal risk. This study will provide more accurate and reliable data for managing pregnant women with UC in Japan.

## Author Contributions

**Tamami Tsuzuki:** conceptualization, methodology, data curation, writing – original draft, writing – review and editing, investigation, visualization, formal analysis, project administration. **Marina Minami:** conceptualization, methodology, investigation, formal analysis, supervision, writing – review and editing, visualization, validation. **Ryuhei Nagai:** supervision, writing – review and editing, methodology. **Yusuke Oki:** supervision, writing – review and editing, investigation. **Hedeyuki Miyachi:** supervision, writing – review and editing. **Masamitsu Eitoku:** supervision, writing – review and editing. **Narufumi Suganuma:** writing – review and editing, supervision, methodology. **Nagamasa Maeda:** writing – review and editing.

## Disclosure

The authors have nothing to report.

## Ethics Statement

The JECS protocol was reviewed and approved by the Ministry of the Environment's Institutional Review Board for Epidemiological Studies (No. 100910001) and the Ethics Committees of all participating institutions.

## Consent

Informed consent was received from all participants.

## Conflicts of Interest

The authors declare no conflicts of interest.

## Data Availability

The data that support the findings of this study are not publicly available due to ethical and legal restrictions in Japan. Data may be available upon reasonable request to the JECS Programme Office, National Institute for Environmental Studies (jecs-en@nies.go.jp).
